# Plants as Model in Biomimetics and Biorobotics: New Perspectives

**DOI:** 10.3389/fbioe.2014.00002

**Published:** 2014-01-30

**Authors:** Barbara Mazzolai, Lucia Beccai, Virgilio Mattoli

**Affiliations:** ^1^Center for Micro-BioRobotics, Istituto Italiano di Tecnologia, Pontedera, Italy

**Keywords:** plant inspired solutions, soft robotics, biomimetics, growing robots, biorobotics

## Abstract

Especially in robotics, rarely plants have been considered as a model of inspiration for designing and developing new technology. This is probably due to their radically different operational principles compared to animals and the difficulty to study their movements and features. Owing to the sessile nature of their lifestyle, plants have evolved the capability to respond to a wide range of signals and efficiently adapt to changing environmental conditions. Plants in fact are able to show considerable *plasticity* in their morphology and physiology in response to variability within their environment. This results in movements that are characterized by energy efficiency and high density. Plant materials are optimized to reduce energy consumption during motion and these capabilities offer a plethora of solutions in the artificial world, exploiting approaches that are muscle-free and thus not necessarily animal-like. Plant roots then are excellent natural diggers, and their characteristics such as adaptive growth, low energy consumption movements, and the capability of penetrating soil at any angle are interesting from an engineering perspective. A few examples are described to lay the perspectives of plants in the artificial world.

## Plants as Models in the Artificial World

The study and the extraction of biological key principles, and their translation in design guidelines for a new generation of robots and technological solutions, have been widely adopted in biomimetics and bionics (Gao et al., 2005; Fratzl and Barth, 2009; Zang et al., 2013). Biologically inspired approaches have been traditionally widely adopted in robotics as well (Ayers and Witting, 2007; Ijspeert et al., 2007; Cutkosky and Kim, 2009; Cianchetti et al., 2011). Robots that implement solutions inspired by nature show capabilities that permit adaptive, flexible interactions with unpredictable environments (Pfeifer et al., 2012; Kim et al., 2013). In the goal to mimic living beings, the different components of an artificial system have to be designed from models of the reference biological systems, in an integrated way, making explicit the general design principles underlying the embodiment. The next generation of robots will be “soft,” because this better allows the interaction with environment, mediated by body, as in natural systems (Tramacere et al., 2012). Advances in “soft technology” will lead to a quantum leap in intelligent robotics. “Soft Robotics” will express at different levels: in sensing (e.g., soft skin to touch, deformable tissue, materials); in movement (e.g., elastic, compliant materials for muscles, tendons; variable compliant actuators, etc.); in interaction (e.g., soft movements, social and cognitive skills); in emotions (e.g., facial expression, body posture, etc.) (Pfeifer and Bongard, 2007).

Traditionally, the most studied models in biorobotics and soft robotics are animals, for a plethora of characteristics or for specific applications (Pfeifer and Bongard, 2007; Mazzolai et al., 2012; Pfeifer et al., 2012; Hawkes et al., 2013; Ma et al., 2013). Rarely, plants have been considered a source of inspiration for robotics or, more in general, for innovative engineering solutions. This is probably due to their radically different operational principles compared to animals and the difficulty to study their movements and features. Nevertheless, owing to the sessile nature of their lifestyle, plants have evolved the capability to respond to a wide range of signals and efficiently adapt to changing environmental conditions. Although plants lack muscles, they generate several different kinds of movements that span from hours or days to milliseconds (Martone et al., 2010; Forterre, 2013). The question of how plants can achieve such a wide range of non-muscular movements has attracted the interest of many scientists, since the pioneering work of Darwin (Darwin, 1880; Hart, 1990; Dumais and Forterre, 2012). An understanding of these non-muscular movements could hold the potential for developments in applied sciences and engineering, especially for the fabrication of novel biomimetic actuation strategies and bioinspired devices (Burgert and Fratzl, 2009).

Plants or plant parts, such as roots or leaves, offer countless cues for making innovation in technology. Some of the main principles studied in the plant roots and, more in general, in plants, in order to be applied to the development of new technologies can be summarized as follows:

- *capacity of growth and movement* in response to external stimuli with high plasticity and morphological adaptation to the environment. This implies a character that can be: nastic (movement that is independent of the spatial direction of a stimulus) or tropic (the response of plant is influenced by the direction of a stimulus); active (live plant cells activate and control the response by moving ions and by changing the permeability of membranes based on potential actions) or passive (movements that are based on dead tissue that is suitable to undergo predetermined modifications upon changes in environmental conditions); reversible or irreversible. This implies the development of new actuation solutions for steering or elongating robotic parts;
- *osmotic actuation*, used for triggering fast movements or driving slow movements in plants. A new generation of actuators, characterized by low power consumption and high energy density can be derived by this fundamental plant property;
- *sensory capabilities* with respect to a wide amount of different physical and chemical quantities in the environment (especially soil). Innovative sensing systems that incorporate at the micro-scale several transduction mechanisms for detecting several parameters, which are intelligently “interpreted” for efficient behavior, are promising technological solutions;
- *growth from the tip* of the root by adding cells and production of lateral hairs, which reduces friction and pressure needed to penetrate the soil. New bioinspired robotic artifacts that grow by adding new materials are emerging;
- *emergent behavior* given by coordination of the root apices of the whole organism toward optimal targets. This research can lead to develop novel methods of collective decision making in decentralized structures with local computation and simple communication.

In the following, we highlight a few examples of solutions inspired by plants, including materials, actuations, and plant root-like robots.

## New Materials and Actuators Inspired by Structures and Motions in Plants

Nowadays one of the major boosts in material science is given by inspiration from nature. The inspiration to natural solution allows the development of new materials with enhanced structural properties, applicable in several fields (including consumer products, automotive, and architecture) and with sensing and actuation capability.

The interesting aspect of natural materials is related to the numerous functionalities that they can exploit (e.g., stability, stiffness, toughness, self-healing, etc.), even if they are based on a limited number of basic components (i.e., cellulose, hemicellulose, lignin, and pectin). This variety of properties is mainly due to the hierarchical organization of such components. Extraction of the principles underlying these properties is one of the key approaches of bioinspired materials.

Of particular interest are those materials and principles that are inspired by plant world, since they are still largely unexploited (except for few well-known cases), and gained attention only recently by material science researchers. Some of the most interesting examples of the recent achievement in this field are reviewed in the following.

The most famous example of technology inspired by plants that is commonly used in many different fields and applications is the Velcro invention. Velcro resulted in 1948 from a Swiss engineer, George de Mestral, noticing how the hooks of the plant burrs (*Arctium lappa*) stuck in the fur of his dog. From this observation, George de Mestral derived the idea of a novel type of zip fastener (Velcro, 1955).

Another interesting industrial solution resulting from the study of material properties in plants is Lotusan^®^, a paint for self-cleaning surfaces. The scientific basis of this invention is the so-called lotus effect: some leaves of water-repellent plants, such as *Nelumbo nucifera* (lotus) and *Colocasia esculenta*, show superhydrophobic and self-cleaning properties, due to the presence of a hydrophobic coating and a multi-scale hierarchical roughness (microstructure formed by papillose epidermal cells covered with epicuticular wax tubules) (Barthlott and Neinhuis, 1997; Bhushan, 2009). The observation that the leaves of the lotus are always clean, despite growing in muddy and stagnant water, led to the production of this new product. Similar surface textures are used to repel dirt or make it easily removed, and have been observed in many other plants and in other systems, such as insect wings (Wagner et al., 1996).

Concerning intrinsic mechanical structural properties, an interesting example of technological solution inspired by plants is given by cellular or sandwich structures consisting of solid shells filled with compliant cellular cores, which have increased resistance against axial buckling while being light weight. These properties are typically required in aerospace applications (Meyers et al., 2008). Particularly interesting are bamboo-inspired fibers and composites for the development of structural engineering materials (Li et al., 1995). Bamboo is one of the strongest natural structural composite materials in which weight to strength ratio is optimized. While having similar chemical composition to wood, bamboo mechanical properties are very different: e.g., tensile strength of bamboo is in the order of 150–520 MPa while in wood it is limited to 30–220 MPa. Such differences are due to the peculiar cellular structure at macro, meso, and micro-scale. At the macro-scale bamboo is a hollow cylinder with many longitudinal nodes, while wood is a solid cylinder. At the meso-scale, bamboo has a non-linear gradient structure in which vascular bundles and thin-walled cells are present, while in wood there are alternated “rings” or layers of spring and summer wood. At the microscale, wood and bamboo fibers have basically analogous multilayer concentric hollow-tubes structure, in which each layer is reinforced with microfibrils. However, the two micro-structures have such differences as their cell wall thickness, layer number, and microfibrillar angle. The relative arrangement of such interlayers and of transition zones in bamboo fibers are known to have a significant effect on fibers properties. By modeling the mechanical behavior of bamboo and by mimicking the arrangement of its fibers, biomimetic fibers realized with composite engineering materials, like glass fiber-reinforced polyester or epoxy resins, have been realized showing improved properties in terms of interlaminar shear strength (Li et al., 1995).

From a structural point of view, another interesting example is given by the pomello, *Citrus maxima*, which shows a peculiar damping system, allowing the fruit to resist to a drop from heights of up to 15 m. In this case the fruit wall, thanks to a complex hierarchical structure consisting of interconnected porous layers combined with branched fiber networks, is able to dissipate more than 90% of the impact energy thus preventing pulp damage (Martone et al., 2010; Thielen et al., 2013). The principles have been exploited in the development of new materials for enhanced crash absorbers, based on metal foams (Fischer et al., 2010).

Bioinspiration from plants is not only restricted to structural materials but also another interesting aspect to study is related to the structure-to-movement relation. For instance, the study of the arrangement of the materials in plant structures and, more in general, of plant biomechanics, as well as of the physical mechanisms used by plants to achieve movements is increasingly recognized as an interesting approach to derive bioinspired devices. The hierarchical structure and orientation of microfibrils of the cellulose layer in the plant cell walls allow passive movements that are mainly driven by changes in environmental conditions. These systems do not require further control and energy supply by the organism once their growth is completed. This plant features make them interesting for biomimetic transfer and offer new insights for designing smart actuators, smart sensors, and new materials, which “respond” to environmental changing conditions (e.g., humidity and temperature).

On these topics, a nice example is given by the Flectofin^®^, a new façade-shading system that is inspired by the open mechanism of the flower of the Bird-of-Paradise (*Strelitzia reginae*). In this flower, the bending mechanism that expose the nectar to birds that land on the perch is used to transfer the pollen to the near flowers, and it is based on a hingeless movement in which an external mechanical force initiates a complex deformation of multiple structural members. In this case, this mechanism has been implemented by means of a glass fiber-reinforced composite, allowing large elastic deformation, driven by variation of temperature that passively can control the façade-shading systems (Lienhard et al., 2011).

A new actuation mechanism based on reversible adsorption and desorption of environmental humidity was recently proposed (Taccola et al., 2013). This device combines the possibility to achieve active and passive actuation with a single composite material, i.e., poly(3,4-ethylenedioxythiophene):poly(styrenesulfonate) (PEDOT:PSS), which is a well-known conjugated conducting polymer that exhibits a unique water absorption capability (due to the hydrophilic PSS). Actuation can be obtained by coupling an ultra-thin film of PEDOT:PSS (a thickness of several hundreds of nanometers) with a passive elastomeric layer (a thickness of hundreds of μm) in a bilayered fashion. If the humidity in the ambient air increases, then the PEDOT:PSS layer adsorbs water vapor and increases in volume, which results in bending of the structure due to the constraints of the passive layer.

The osmotic principle, so cleverly harnessed by plants to produce their movements, has also been considered for actuating several artificial systems (Sudaresan and Leo, 2008; Piyasena et al., 2009). In particular, an osmotic actuator was proposed to steer the tip of a mechatronic system inspired by the apex of the plant roots (Mazzolai et al., 2011) for soil exploration and monitoring. Furthermore, a dynamic model of the osmotic actuation concept was developed based on elements that remember key basics in plants, such as an osmotic membrane, an actuation chamber that contains the osmotic agent and both a rigid and a deformable boundary, and a solute reservoir chamber (Sinibaldi et al., 2013).

## Robotic Solutions Inspired by Plant Roots

The distinctive living form that confers a plant root the power to penetrate and explore soil at first instance concentrates in the root apex. Briefly said, root apex grows adding new cells, which move from meristem to elongation zone, where they expand axially because of the water absorbed by osmosis and the directional loosening of the cell wall. This action creates an interface between the soil and the root apex, and allows the root to penetrate the soil with only a small part of its body (the apex), while the remainder of the structure is stationary and in contact with the soil (the mature region). Hairs generating at root fringe anchor the structure to the soil and increase the available surface for water and nutrients uptake. Cell division and morphology are influenced by the surrounding environment, and so the growth process at the tip enables the root to adapt to the environmental conditions such as soil texture and mechanical impedance (Dexter, 1987).

Growth kinematics and morphological features were extensively investigated (Bengough et al., 2011) through observations of living roots in various environmental conditions, together with modeling and simulation (Clark et al., 2008). Cells expansion, elongation, sloughing, and differential elongation are therefore all well-known strategies concentrated at root tip level, that are used to follow low impedance pathways in the soil.

Taking advantage of strategies to penetrate and explore soil as well as to maintain good performance in terms of energy efficiency are totally new research goals in bioinspired robotics that are attracting different scientific communities like biology, physics, and material science. A first multidisciplinary research effort in this direction is focusing on robotic solutions that are called “PLANTOIDS,” which are robotic systems equipped with distributed sensing, actuation, and intelligence to perform soil exploration and monitoring tasks.

In such complex process of inventing efficient mechanisms for soil penetration, the first important strategy to be deciphered is related to the way dynamical friction develops on a limited part of the root body (tip level) while the rest is firm and anchored to the soil. An important aspect of growth regards the sloughing of cells from the tip, and to this regard a simple but effective artificial method was investigated (Sadeghi et al., 2013). A soft and flexible tubular skin was stored inside a rigid shaft so that it could traverse the hole to the external surface of the shaft. The outward movement of skin from the tip was activated by a motor on top of the shaft connected to the skin, and it provided a low-friction interface between the shaft and soil. A second aspect was also implemented: to mimic the anchoring achieved by the natural root hairs that grow laterally to its wall, artificial soft hairs were integrated on the external part of the skin in contact with the soil. The robotic system was able to penetrate a granular substrate using the movement of the soft skin without adding any external force. It was demonstrated that upon increasing the hair density, the efficiency of the penetration increased to approximately 30% and the axial penetration force decreased owing to the skin movement.

However, the proposed sloughing system alone is not able to provide friction reduction for an artificial root; externally the skin interaction with soil along the shaft is stationary, but internally the relative movement of the skin and the penetrating shaft can produce a resisting friction force which increases by increasing the depth of penetration. Concentrating more on the root-growth approach at the apical region, the “elongation from the tip” (EFT) strategy was studied with artificial systems (Tonazzini et al., 2013). It is noteworthy to mention that the amount of penetration energy required for a rigid probe with EFT was less than the energy required for the entire artificial root body insertion in granular media (reductions went from approximately 20 to 50%).

On the basis of these results, a system that grows at the apical area by the addition of new material was recently developed (Sadeghi et al., submitted for publication), which represents a totally new approach in robotics, embodying *artificial growth*. The system embeds a *growing zone* and a stationary *mature zone*. The *mature zone* consists of a hollow, tubular structure that allows the transfer of new material from a spool (external to the robotic root) and power to a *growing zone*. The growing zone is based on an additive layering mechanism that generates a force for penetration into the soil and that transfers a material (which is in the form of a filament) from the external system to the head (or tip). The *growing* capability at the tip therefore is achieved by means of an additive manufacturing technique, and the layer-by-layer deposition creates the *mature zone*, which uses a tubular body as a support structure that moves axially inside the mature zone in a passive manner. The new material is distributed on the surface of the deposition head, in the *growing zone*.

**Figure 1:**
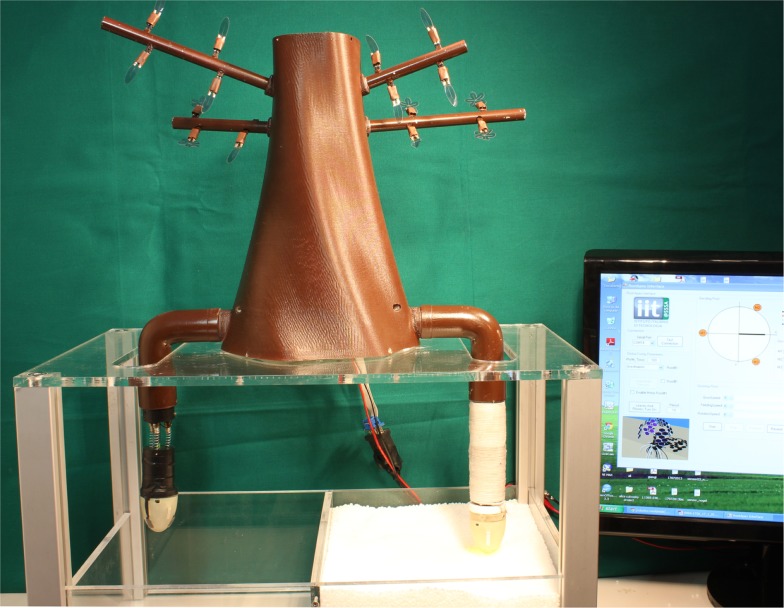
**The PLANTOID robot**. It integrates two roots that implement the elongation movement and the bending motion with a sensorized tip, respectively. In this prototype, the tip connected to the bending root integrates sensors for touch, humidity, temperature, and gravity. The branches integrate artificial leaves based on PEDOT:PSS material that move in response to changes in environmental humidity.

From these initial achievements, the basic principles learned from the natural root behavior could be validated and, by adding flexibility, the system could be designed to change the direction and navigate around the obstacles. In Figure 1, a first prototype of the PLANTOID is shown. The system has a main trunk to which two roots are connected. One root embodies artificial growth like explained above and it elongates and penetrates the soil by an additive process of material (Sadeghi et al., submitted for publication). Another root integrates the bending capability in three directions and a sensory system for temperature, humidity, gravity, and touch.

The approach to take inspiration to Plant Kingdom and to translate plant features in artificial solutions, especially in the robotic field, is moving its first steps. Many of the structural, functional, and physiological properties of plants and plant parts represent a revolutionary source of inspiration because they are based on evolutionary strategies aimed at reducing energy consumption and optimizing the use of local resources. This results, for example, in plant structures that exploit the environmental energy to move or implement efficient motion strategies. Therefore, the first lesson that we must take from plants is to watch them with different eyes.

## Conflict of Interest Statement

The authors declare that the research was conducted in the absence of any commercial or financial relationships that could be construed as a potential conflict of interest.
